# Evidence from Toxicology: The Most Essential Science for Prevention

**DOI:** 10.1289/ehp.1509880

**Published:** 2015-06-19

**Authors:** Daniele Mandrioli, Ellen Kovner Silbergeld

**Affiliations:** 1 Department of Environmental Health Sciences, Johns Hopkins Bloomberg School of Public Health, Baltimore, Maryland, USA; 2 Cesare Maltoni Cancer Research Center, Ramazzini Institute, Bologna, Italy

## Abstract

**Background:**

The most essential goal of medicine and public health is to prevent harm (*primum non nocere*). This goal is only fully achieved with primary prevention, which requires us to identify and prevent harms prior to human exposure through research and testing that does not involve human subjects. For that reason, public health policies place considerable reliance on nonhuman toxicological studies. However, toxicology as a field has often not produced efficient and timely evidence for decision making in public health. In response to this, the U.S. National Research Council called for the adoption of evidence-based methods and systematic reviews in regulatory decision making. The U.S. Environmental Protection Agency (EPA), the Food and Drug Administration (FDA), and the European Food Safety Agency (EFSA) have recently endorsed these methods in their assessments of safety and risk.

**Objectives:**

In this commentary we summarize challenges and problems in current practices in toxicology as applied to decision making. We compare these practices with the principles and methods utilized in evidence-based medicine and health care, with emphasis on the record of the Cochrane Collaboration.

**Discussion:**

We propose a stepwise strategy to support the development, validation, and application of evidence-based toxicology (EBT). We discuss current progresses in this field produced by the Office of Health Assessment and Translation (OHAT) of the National Toxicology Program and the Navigation Guide works. We propose that adherence to the Cochrane principles is a fundamental prerequisite for the development and implementation of EBT.

**Conclusion:**

The adoption of evidence-based principles and methods will enhance the validity, transparency, efficiency, and acceptance of toxicological evidence, with benefits in terms of reducing delays and costs for all stakeholders (researchers, consumers, regulators, and industry).

**Citation:**

Mandrioli D, Silbergeld EK. 2016. Evidence from toxicology: the most essential science for prevention. Environ Health Perspect 124:6–11; http://dx.doi.org/10.1289/ehp.1509880

## Introduction

The most essential goal of medicine and public health is to prevent harm (in the words of Hippocrates, *primum non nocere*). This goal is only fully achieved with primary prevention, which requires us to identify harms prior to human exposure. Toxicology, almost always involving nonhuman subjects, is the main source of such information. Bioethical principles of human subjects research have developed in response to several examples of morally reprehensible research involving humans over the past 70 years (Josefson 2001; Katz et al. 2006) that prohibit the deliberate testing of humans for the purpose of establishing toxicity without expected benefit to the subjects of such testing (Silbergeld et al. 2004).

For preventing harms, we need to have reliable and sufficient evidence of safety for chemicals, drugs, and food prior to permitting human exposure, particularly in our chemical world, with tens of thousands of chemicals in commerce and the environment. This ethic underlies the establishment of many regulations and guidance by governments and international institutions requiring preapproval testing of substances developed for their biological activity, such as pharmaceuticals, in order to assess likely benefits and harms prior to testing in humans. The same principle is applied for testing other chemicals developed for their toxic properties, such as pesticides. For other chemicals produced by industry, the situation is less consistent (Silbergeld et al. 2015). For the many chemicals that are already on the market, nonhuman toxicological evidence can support prudent actions to reduce exposures without the delays and human costs of awaiting evidence from observational studies.

Despite its crucial position in science-based public health policy, toxicology as a field has often failed to efficiently produce timely information for decision making and prevention of harms (EEA 2013). As a consequence, policy making in environmental and occupational health and in drug and product safety lags far behind the need for prevention of harms. There are many reasons for this, including the failure of current methods in applying toxicological information to resolve controversies among stakeholders (Silbergeld et al. 2015). Part of this is certainly related to the economic and political importance of the issues for which toxicological information is generated, such as drug and chemical approvals and legally binding standards for air and water. However, toxicology as a field contributes to its own failures to generate information expeditiously and to respond to controversies through its lack of systematic methods and evidence-based principles similar to those that have been successfully applied to resolve controversies and reach decisions in other fields related to public health.

The wake-up call for the field of toxicology came with the recent U.S. National Research Council (NRC) recommendation to the U.S. Environmental Protection Agency (EPA) for the adoption of evidence-based methods, similar to those widely used in medicine and health care, in its assessments of chemical hazards and risks. The NRC report (NRC 2014a) included a strong critique of the current reliance on nontransparent processes such as “weight of evidence.” The U.S. EPA (Cogliano 2014; NRC 2014a), the Food and Drug Administration (FDA 2009), and the European Food Safety Agency (EFSA 2010) have made public commitments to the development and application of systematic methods for evaluating evidence from the toxicological sciences. The International Agency for Research on Cancer (IARC) has begun to utilize these methods in its monographs on carcinogens (Hamra et al. 2014). With these developments, there is now wider acceptance that evidence-based methods, including systematic reviews, is “the road worth taking” for toxicology (Silbergeld and Scherer 2013). Less well understood is what this acceptance entails. In this commentary, we define and discuss both the core principles and methods of evidence-based practice that are applicable to toxicology, with specific reference to the ones developed and used by the Cochrane Collaboration, an international not-for-profit organization preparing, maintaining, and promoting the accessibility of systematic reviews of the effects of health care (Cochrane Collaboration 2015a). Using a comparison between evidence-based practice and current practices in toxicology, we examine the differences, limits, and advantages of both principles and methods for toxicological research and application to public health policy.

## Discussion

*Toxicology: a matter (not just) for experts*. The importance of toxicology is widely recognized and accepted in public health policy. However, the reliability and validity of many toxicological methods—from study design to statistical analyses—have been challenged. These limitations have significant impacts for both improving and protecting health. Recent reviews have demonstrated the low predictive value of preclinical testing in identifying novel pharmaceutics likely to have therapeutic benefits, as well as in detecting potential adverse effects early in drug development (Krauth et al. 2014). These failures may result in costs of millions of dollars in development as well as harms to patients (Kola and Landis 2004). For nonpharmaceutical chemicals, including food additives, current toxicological methods and practices do not resolve controversies because of their nontransparent procedures and potential for conflict of interest. Too often, decisions are based on information provided by and evaluated by parties with financial ties to the products without public disclosure (Abdel-Sattar et al. 2014; Neltner et al. 2013). As a consequence, debates over the hazards of many of these agents—already in production and use—go on for decades, with controversies among regulatory agencies within and among countries, states, and stakeholders. In a recent review, we also observed that the assessment of new chemicals prior to production relies heavily on nonvalidated methods and nontransparent data submissions (Silbergeld et al. 2015).

Despite the increasing resources devoted to toxicity testing of drugs and chemicals in terms of animals, time, and expertise, the pace of regulatory decision making by agencies such as the U.S. EPA is best described as glacial. Recently, the National Academy of Sciences (NAS) was called on by the U.S. Congress to review National Toxicology Program (NTP) Report on Carcinogens listings of styrene and formaldehyde as carcinogens (NRC 2014b, 2014c). These two major industrial chemicals are produced and used in many countries at a level of millions of tons per year, and panels with different experts have expressed divergent opinions on the hazards of these two chemicals (NRC 2014b, 2014c). Toxicological information from the NTP and the Ramazzini Institute on the hazards and risks of these two chemicals has been publicly available for decades (Conti et al. 1988; NTP 2011; Soffritti et al. 2002), yet definitive regulatory action has been delayed. Regulatory delays concerning styrene and formaldehyde, as well as delays reaching decisions with other chemicals, have prevented actions to reduce harms resulting from continued exposures, an example of what the European Environment Agency described as “late lessons from early warnings” (EEA 2013). In many cases there are no early warnings because most chemicals are not tested before marketing or are marketed with insufficient evidence of safety. This still happens (for example in the United States and China) in full compliance with current chemical regulatory policies such as the Toxic Substances Control Act of 1976 (Silbergeld et al. 2015). A tragic example of this practice is 4-methylcyclohexanemethanol. The accidental release of this chemical in West Virginia led to the shutdown of drinking water for > 700,000 people because health hazards associated with its use were largely unknown (Manuel 2014).

The limits of the discipline of toxicology and the delayed promulgation and application of effective regulatory policies based on the use of toxicological principles contributed to the impetus for the precautionary principle largely in order to empower timely preventive actions (Collegium Ramazzini 2004; EEA 2013). The increasing public pressure for more rapid action to protect public health and the environment has supported policies that reduce the requirements for full information. In fact the precautionary principle definition promulgated in 1992 by the United Nations (UN) Conference on Environment and Development states

In order to protect the environment, the precautionary approach shall be widely applied by States according to their capabilities. Where there are threats of serious or irreversible damage, lack of full scientific certainty shall not be used as a reason for postponing cost-effective measures to prevent environmental degradation. (UN 1992)

But the precautionary principle does not remove the need for toxicological evidence for “threats of harm” and does not help decisions that require quantitation of harm such as most air and water quality standards. Others are placing hope in alternative methods, such as “Tox21,” where high-throughput molecular-based systems are proposed to shift the assessment of chemical hazards away from traditional experimental animal toxicology studies to methods that reduce time and the burdens on animal use in experimentation by substituting mechanism-based *in vitro* assays and *in silico* assessments (Tice et al. 2013). The jury is still out on the utility of these methods to provide sufficient evidence of safety for either pharmaceutics or chemical regulation (Schmidt 2009), and the Tox21 program “will likely take decades to fully achieve its goals” (Tice et al. 2013). In the meantime, other policies, such as the European Union (EU) REACH chemical regulation (ECHA 2015), have attempted to reduce the “burden of proof” on governments to meet the demand for information by placing responsibility on industry to generate toxicology data under the principle of “no data, no market” (Silbergeld et al. 2015). But the quality of these toxicological data and the methods used for their evaluation are other concerns, as discussed below.

Why is toxicology failing? The methodological failures in current nonhuman testing described by Hooijmans and Ioannidis are endemic to the field of toxicology (Hooijmans and Ritskes-Hoitinga 2013; Ioannidis et al. 2014), including inappropriate study designs and inadequate statistical analyses. New tests have been adopted, such as structure–activity analysis and many *in vitro* methods, without appropriate validation (Knudsen et al. 2011), and the process of updating methods is extremely slow. In many respects, toxicology is its own worst enemy. The causes of its malaise are many but not hard to identify. The most critical afflictions of toxicology at present relate to its lack of principles commonly accepted as essential to evidence-based practice, an aversion to transparency, and persistent adherence to nonsystematic methods. As a consequence, toxicology in practice demonstrates little consistency in terms of even assembling the relevant literature, with no clear methods for screening this literature or for extracting and evaluating information in order to objectively test its reliability as evidence. As discussed below, all of these steps precede the integration of evidence for decision making.

Of greatest concern, toxicology has failed to adopt clear principles that could enhance its acceptability. Chief among these is the continuation by toxicology to extensively rely upon “expert judgment.” This concept is embedded in nontransparent and vague principles and practices such as “weight of evidence,” which was recently strongly criticized by the NRC (2014a). Douglas Weed succinctly characterized this term in his 2005 review, in which he concluded that it is not well-defined nor does it refer to a consistent or transparent methodology (Weed 2005). Some of the “principles” often cited in toxicology as indicative of reliability and quality are of unproven relevance in ensuring the reliability and quality of evidence derived from toxicological studies. For example, the Good Laboratory Practices (GLP) code (OECD 1998) is a recipe for keeping adequate records, not for ensuring appropriately designed or valid studies. The Klimisch Score (Klimisch et al. 1997), currently widely used for assessing the reliability of toxicological studies, overvalues compliance with GLP and guidelines and fails to address some of the most important criteria for assessing quality of studies, such as the validity and relevance of the study design, statistical rigor, and attention to sources of bias (Ågerstrand et al. 2011; Myers et al. 2009).

The largest elephant in the room is the failure of toxicology as a field to examine its own biases in terms of conflicts of interest (LaDou et al. 2010). Bero and others have demonstrated that the source of the piper’s pay in research, from clinical trials to tobacco studies, introduces a predictable risk of bias in results and conclusions (Barnes and Bero 1998; Bero et al. 2007; Lundh et al. 2012). For this reason, conflict of interest (COI) was recently proposed as an independent item in the assessment of risk of bias in the Cochrane review process (Bero 2013). Several analyses suggest that the same topic is also important in toxicology and needs more examination as well (Barnes and Bero 1998; Neltner et al. 2013). One group working on evidence-based toxicology in The Navigation Guide already embeds COI as an item in its risk of bias assessment (Woodruff and Sutton 2014).

Toxicology also has a history of service to private interests, which indicates a particular need to evaluate sources of funding as related not only to study bias but also claims of evidence-based practices from interested stakeholders and their consultants (Ashford et al. 2002; Denison 2014; EBTC 2015; Guzelian et al. 2005; Pearce et al. 2015; ToxStrategies 2015). The case of the Klimisch Score is paradigmatic: It was proposed by industry scientists of BASF and has been widely adopted by regulators, despite its lack of validation or relevance to any systematic assessment of the quality of the studies (Klimisch et al. 1997). There are other examples of the same pressures from industry and acquiescence by regulators in terms of the test methods of the OECD chemicals program that now form the basis for the EU REACH program (Ponti et al. 2014).

*A call to (systematic) action*. Calls for the adoption of systematic methods to support the generation of evidence in toxicology are not new, and there are several organizations claiming to use “evidence-based toxicology,” although there is no common accepted definition of this term (Silbergeld and Scherer 2013). At this point in time, a wide community of participation is highly recommended, with some common understanding of what this term implies. In this commentary, we recommend that those interested in evidence-based toxicology, especially regulators, can usefully learn from experience in the first “evidence-based” fields, medicine and health care, which is embodied most fully by the international Cochrane Collaboration (Cochrane Collaboration 2014). Cochrane principles and methods were considered radical and highly disputed when presented several years ago (Dickersin and Manheimer 1998), and thus we can expect a similar context for the development of systematic methods in toxicology (Silbergeld and Scherer 2013). However, we may be able to shorten this initial “postnatal” period by learning from the past. The Cochrane Collaboration has worked for > 20 years to develop both principles and methods. Their systematic methods and reviews are internationally considered as the gold standard in medicine and health care because of their demonstrated value and reliability through decades of development, validation, application, and continuous improvement (Jørgensen et al. 2006; Tovey 2014). We present the case that the new field of “evidence-based toxicology,” which at present has multiple meanings and groups working on methodologies, can learn from both the principles and practices of systematic reviews within the Cochrane Collaboration to develop consensus approaches that can also be internationally accepted. We also consider the additional benefit that the introduction of evidence-based methods in toxicology will provide by enhancing the scientific development and the quality of studies in the field, in a manner similar to the experience in clinical trials in medicine.

*Learning from Cochrane: principles first*. Seventy years ago, problems similar to the ones that toxicology is now facing characterized the challenge of obtaining reliable evidence for medical practice. The use of evidence-based approaches first started with the need for the postwar UK National Health System to be able to reliably evaluate evidence of demonstrably efficacious interventions and treatments in order to approve payment. This was the birth of evidence-based medicine (Dickersin and Manheimer 1998). From this very practical beginning, the Cochrane Collaboration grew into an essential global partner in ensuring evidence-based practices and decision making in health. Its methods now cover diagnostic and test methods as well as interventions and methods of outcome assessment (Cochrane Collaboration 2015b).

Sir Archie Cochrane’s medicine can assist toxicology as well by bringing this essential science into harmony with the principles and practices of evidence-based medicine. As a first step in developing evidence-based toxicology, the principles of evidence-based medicine can be adopted straight from the Cochrane prescription. These principles have been proven solid and reliable, even when addressing controversial themes (Gøtzsche and Jørgensen 2013; Jefferson et al. 2010). As shown in Appendix 1, these principles state the prerequisites for ensuring that work in Cochrane will produce reliable evidence for decision making (Cochrane Collaboration 2015c). These principles include the identification and reduction of bias (i.e., factors that introduce systematic error and otherwise reduce confidence in results) and methods of work that enhance the achievement of this goal through transparency at all stages, open collaboration and access, validation and improvement of methods, and continuous updating of reviews. These principles consider the legitimate interest of all the stakeholders (researchers, consumers, regulators, and industry), where collaboration and public health interest prevail over single interests.

Many toxicologists at this time do not abide by these principles, as is clear from a recent position statement by a group of industry, government, and academic representatives, “An Appeal for the integrity of Science and Public Policy” (Gori et al. 2015), in which they argue that the “rules of evidence of the scientific method” are to be preferred in establishing decisions regarding assurance of safety and prevention or risk. The appeal defines the scientific method without including the principles of transparency, participation, or adherence to the identification of sources of bias, including conflict of interest. This has been one source of toxicology’s present difficulties and a major contributor to the difficulty of resolving controversies.

*Learning from Cochrane: method, follow*. In terms of methods, many of those already developed and validated by the Cochrane Collaboration can be adopted, some will require modification, and some adjustments specific to toxicology may require the development and validation of new formulations to achieve an evidence-based approach.

The Cochrane Collaboration has developed protocols to guide steps in the process of systematic reviews that have been demonstrated to produce useful and reliable information. These protocols are readily adaptable to toxicology: They include clear formulation of the problem to be reviewed; comprehensive and explicit strategies for identifying sources of information; attention to all sources of bias, including inadequate study designs and unvalidated or inappropriate methods of generating and analyzing information; and public disclosure of financial conflicts of interest.

Differences between toxicology and evidence-based practice are illustrated in Table 1.

**Table 1:** Methods: toxicology vs. evidence-based toxicology.

Toxicology	Evidence-based toxicology
Unclear answers to unclear questions	Clear formulation of problem (PECOTS)
Noncomprehensive research strategy	Comprehensive research strategy
Nontransparent methods	Transparent methods
Unvalidated methods	Requirement to validate methods prior to use
Inadequate study design (e.g., effect size, expected variability)	Adequate study design
No or inconsistent assessment of risk of bias	Assessment of risk of bias
Inadequate or no statistical modeling	Appropriate statistical modeling based on appropriate study design
Conflict of interest usually not disclosed	Conflict of interest disclosed
Unvalidated or irrelevent guidelines for practice (Klimisch Scores, Good Laboratory Practices)	Specific evaluation of risk of bias and compliance with evidence-based practice
PECOTS: populations, comparators, outcomes, timings, and settings of interest.	

Well-validated methods and practices of systematic reviews, as developed by the Cochrane Collaboration, can be largely translated to toxicology (Rooney et al. 2014):

Clarity in formulation of the problem: defining populations, exposures, comparators, outcomes, timings, and settings of interest (PECOTS)
Transparent and replicable processes for research strategy
Transparent methods of data extraction and presentation
Validation of all methods and criteria in terms of relevance to reducing bias
Comprehensive assessment of risk of bias (study design, appropriate statistical analyses, conflict of interest)
Transparent criteria for determining if data integration is appropriate and conducting data integration, such as meta-analysis.

However, challenges in developing evidence-based methods specific for toxicology will also require new adequate methods that cannot be directly derived from Cochrane. For example, while sharing common problems (and perhaps some common solutions), nonhuman preclinical studies and toxicology tests require some different methods and policies because of their differing purposes: Preclinical studies investigate efficacy (benefits), and toxicology investigates safety (harms) (Krauth et al. 2013). There are particular aspects of nonhuman studies that will require investments and efforts to develop methods, including:

Attention to external validity of nonhuman toxicity tests for inferring risks to humans
Challenges to integrating information: *a*) dealing with the diversity of nonhuman species currently used in toxicity tests as well as the use of *in vitro* systems, organotypic cultures, transformed cell lines, and *ex vivo* preparations; and *b*) assessing the validity of “toxicity pathway” studies
Determining the contribution and value of mechanistic studies to overall evaluation of evidence
Moving beyond harms: generating evidence to support decisions for setting regulatory standards (i.e., dose response).

The NTP Office of Health Assessment and Translation (OHAT) *Handbook for Conducting a Literature-Based Health Assessment Using OHAT Approach for Systematic Review and Evidence Integration* (NTP-OHAT 2015) and the “Navigation Guide Systematic Review Methodology” (Woodruff and Sutton 2014) are two important efforts to translate and embed many of the above-mentioned Cochrane ingredients in toxicology. There is also ongoing work for implementing specific methods for integrating and grading the quality of evidence in toxicology (Rooney et al. 2014). Particularly relevant is the implementation of GRADE (Grades of Recommendation, Assessment, Development, and Evaluation), a system for grading the quality of evidence used by several organizations worldwide (including Cochrane Collaboration and the World Health Organization), with specific scales that should be tailored for rewarding sensitivity of the studies to harm detection and prevention (the main outcomes of interest for toxicology), rather than efficacy (the main outcome of interest of clinical medicine and preclinical studies) (Guyatt et al. 2008). Harmonization and upgrades will be necessary following the first attempts of systematic reviews in toxicology, and adherence to common principles and methods will be the first necessary step toward the application of evidence-based approaches in toxicology.

## Conclusions

Improving the methods of generating systematic evidence from toxicology will not only clarify and expedite the processes of decision making but will also enhance the international acceptability of a common evidence base that can be fitted into national policies (NRC 2014a). This is an important and significant challenge to our field; however, we come to this challenge on the shoulders of considerable achievements in developing and applying systematic methods in other relevant fields, such as the ones obtained by the Cochrane Collaboration in its work related to evidence-based medicine and health care. As with experience in Cochrane, our dedication to generate systematic evidence by ensuring comprehensive and objective analyses will improve the process of decision making, thereby preventing harms, increasing public confidence, and reducing costs. Moreover, success in this effort will improve and strengthen the science of toxicology, just as adoption of the systematic approach to evaluating information from clinical trials has resulted in the adoption of more reliable methods, with lower risk of bias and more predictive value.

Appendix 1 **Cochrane’s Principles (Cochrane Collaboration 2014)**

Collaboration: by fostering global cooperation, teamwork, and open and transparent communication and decision making.
Building on the enthusiasm of individuals: by involving, supporting, and training people of different skills and backgrounds.
Avoiding duplication of effort: by good management, co-ordination, and effective internal communications to maximise economy of effort.
Minimising bias: through a variety of approaches such as scientific rigour, ensuring broad participation, and avoiding conflicts of interest.
Keeping up to date: by a commitment to ensure that Cochrane Systematic Reviews are maintained through identification and incorporation of new evidence.
Striving for relevance: by promoting the assessment of health questions using outcomes that matter to people making choices in health and health care.
Promoting access: by wide dissemination of our outputs, taking advantage of strategic alliances, and by promoting appropriate access models and delivery solutions to meet the needs of users worldwide.
Ensuring quality: by applying advances in methodology, developing systems for quality improvement, and being open and responsive to criticism.
Continuity: by ensuring that responsibility for reviews, editorial processes, and key functions is maintained and renewed.
Enabling wide participation: in our work by reducing barriers to contributing and by encouraging diversity.
